# Mastering online ophthalmology exams: essential tips for success

**DOI:** 10.56920/cehj.861

**Published:** 2025-08-05

**Authors:** Ashok Kumar, Essam Eltoukhy, Eduardo Mayorga, Henry E Nkumbe

**Affiliations:** 1 Grover Chairman: Vision Eye Centres & Sr Consultant: Sir Ganga Ram Hospital New Delhi, India.; 2 Professor of Ophthalmology: Cairo University Chairman of the Egyptian Board of Ophthalmology. Director of Training: Arab Board of Health Specialities.; 3 Senior Consultant in Medical Education: Hospital Austral, Buenos Aires, Argentina.; 4 CEO and Chief of Retina and Vitreous Department: Magrabi ICO Cameroon Eye Institute, Yaounde, Cameroon.

**Figure:**
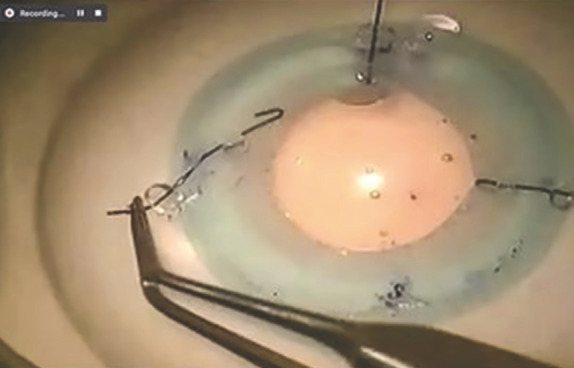
Testing knowledge of surgical devices and techniques.

© CEHJ CC BY-NC-SA 4.0

Approaching an online exam may be different from previous exam experiences, and involves both knowledge and self preparation.

Examiners and a top-performing candidate have contributed these tips.

## Before you book your exam date:

- Are you at the right stage of your training to take the exam? Speak to your supervisors and mentors if you are not sure.
- Do you have enough time before your chosen date?
- **TOP TIP:** It may be helpful to focus on one exam at a time, so you have enough time to prepare for each one.

## Creating a study plan

- Start by visiting the relevant website to familiarise yourself with the content that will be tested. Review the recommended reading list.
- Identify how many hours you can dedicate to exam preparation each week, and how many hours you have available in total before the exam date.
- Create a structured study plan, allocating sufficient time to each topic area.
- Familiarise yourself with the exam format and the technology requirements to take the exam. What platform is used by the exam host? Is there proctoring (exam monitoring), and how does it work online? Are questions multiple choice, or are there open book / short answer elements?

## What to study

- Begin by focusing on the basic sciences which form the foundation for understanding clinical ophthalmology concepts, particularly anatomy, physiology, and optics.
- Next, study clinical ophthalmology (pathology and clinical management) using illustrated texts, clinical atlases, and online resources (including videos, quizzes, and updated practice guidelines). Many exam questions use clinical pictures to test your diagnostic examination and recognition skills.
- Throughout the preparation period, you may need to periodically revisit the basic sciences to reinforce your understanding of fundamental concepts.

## Practicing for the exam

An essential component of preparation involves practising with questions in the same format as your chosen exam. It's crucial to not just answer questions, but to read additional material relevant to the question.

- Make sure you understand the exam format. Review the structure, question types, and timing.
- Practicing regularly under timed conditions boosts confidence, highlights weak areas, and improves speed and accuracy, ultimately increasing the chances of success.
- Understand the marking scheme for the exam. Is there negative marking?
- Check when results are to be announced, and ensure it aligns with your training pathway.
- Engage in active learning – a technique that gets you to learn through activities and discussions, rather than just through studying alone. For example, you could create your own questions in the format of your chosen exams and exchange them with colleagues, then discuss your answers.

## Technology for the exam

Taking an online exam means a crucial element of preparing is to ensure you are familiar with the technology you will be using to take the exam, and ensuring you know how the platform works. Create a checklist in plenty of time, to be confident in the process on the day.

- Do I need to download any software?
- Are there minimum system requirements?
- What are rules on reasonable adjustments during the exam, such as coloured backgrounds, additional time, or rest breaks?
- Is there a help desk, in case I run into problems on the exam day?
- Who does ID checks?Do I need a webcam?

## Resources to support your learning

- Consider using the resources (including self- assessment) on AAO's One Network. Access is free to ophthalmologists in low- and lower-middle-income countries (bit.ly/44th7pU)
- Quizzes and case studies are available free of charge at CyberSight.org
- Although expensive, question banks such as the BCSC Self-Assessment from AAO and OphthoQuestions can be helpful. Practice these questions first in tutor mode, then progress to timed exam mode.

## The day before the exam

- Be sure to have a good night's sleep, so that you are well rested on the day.
- If travelling to an exam centre, plan in advance so that you arrive early and avoid rushing.
- Approach the exam with a positive mindset, reminding yourself that you're in it to succeed.

## On the day of the exam

- Time yourself, set milestones to track your progress, and avoid spending too much time on difficult questions – move on and come back to them later if needed.
- Remember to answer all questions, even if you're unsure of the answers.

After the exam, treat yourself to something enjoyable while you wait for the results!

**Figure:**
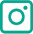
@ophthalmology.foundation

**Figure:**
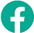
@Ophthalmology Foundation

**Figure:**
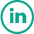
@Ophthalmology Foundation

**Figure:**
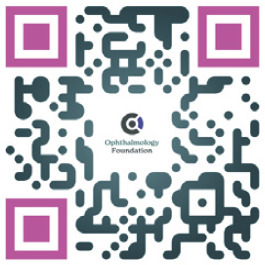


*The content of this page is supported by the Ophthalmology Foundation*

